# The Activation Effects of Low Level Isopropyl Alcohol Exposure on Arterial Blood Pressures Are Associated with Decreased 5-Hydroxyindole Acetic Acid in Urine

**DOI:** 10.1371/journal.pone.0162762

**Published:** 2016-09-13

**Authors:** Zhiqiang Zhao, Xinxia Liu, Xiumei Xing, Yao Lu, Yi Sun, Xiaoyan Ou, Xiaolin Su, Jun Jiang, Yarui Yang, Jingli Chen, Biling Shen, Yun He

**Affiliations:** 1 Guangzhou Key Laboratory of Environmental Pollution and Risk Assessment, School of Public Health, Sun Yat-sen University, Guangzhou, Guangdong, China; 2 Prevention and Control Center for Occupational Diseases, Zhongshan Center for Disease Control and Prevention, Zhongshan, Guangdong, China; Universidade Federal do Rio de Janeiro, BRAZIL

## Abstract

**Purposes:**

The objectives of this paper are to study the impact of low level isopropyl alcohol exposure on blood pressure and to explore its potential mechanism.

**Methods:**

This cross-sectional study was based on a prospective occupational cohort in south China, which focusing on occupational risk factors related cardiovascular health problems. A total of 283 participants (200 low isopropyl alcohol exposed workers and 83 controls) was finally enrolled in this study. Linear regression models were used to analyze the relationship between arterial blood pressures and low level isopropyl alcohol exposure. We used mediation method to explore possible mediated roles of neurogenic factors.

**Results:**

Systolic blood pressure (SBP, 123±10 vs. 118±11), diastolic blood pressure (DBP, 79±7 vs. 74±7) and mean blood pressure (MBP, 93±8 vs. 89±9) were different between the exposed group and the control group (*p < 0*.*01*). After adjusting for covariates, the difference was still significant. Besides, isopropyl alcohol and smoking had an interactive effect on DBP and MBP (*p < 0*.*05*). Furthermore, we observed a mediated effect of 5-hydroxyindole acetic acid (5-HIAA) on isopropyl alcohol exposure induced arterial blood pressure increase, which accounted for about 25%.

**Conclusions:**

Our results suggest that low level isopropyl alcohol exposure is a potential risk factor for the increased arterial blood pressure and 5-HIAA partly mediates the association between low level isopropyl alcohol exposure and arterial blood pressures.

## Introduction

Isopropyl alcohol (isopropanol) is a kind of clear, colorless, flammable organic solvents. It is miscible with water, benzene, chloroform, ethanol or glycerol [1]. Isopropyl alcohol and its raw materials have widely application in pharmacy, coating and printing. Often,isopropyl alcohol can be found in many household detergents, cosmetics, disinfectants and paint thinner. In 2012, there were 9833 Americans exposed to rubbing alcohol products and 4456 were exposed to isopropyl alcohol containing cleaning products or hand sanitizers [2]. Thus, the effects of environmental and occupational isopropyl alcohol exposure become urgently need in-depth studies in future [1,3].

Isopropyl alcohol can be absorbed through digestive tract [4–6], respiratory [7], skin [8,9] and the placenta [10]. It is mainly metabolized by the liver alcohol dehydrogenase (ADH). Its main metabolite is acetone [11–13]. The elimination of isopropyl alcohol is mainly through the kidneys, its half-time is 2.8 ~ 8.0 hours [13,14]. The half-time of acetone is 7.7 ~ 27 hours, a bit slower than isopropyl alcohol [14,15].

Isopropyl alcohol exposure mainly influences the nervous system [16–19], but it may also have reproductive development toxicity [20,21], genetic [22] and teratogenic toxicity [23] and immune toxicity [24,25]. In addition, isopropyl alcohol exposure can also affect the cardiovascular function, but most of these studies were on the basis of high level isopropyl alcohol exposure and the cardiovascular effects were mostly observed under acute exposure or poisoning conditions. Although it is clear that high concentration of isopropyl alcohol exposure can lead to hypotension [26–28], shock [29] and arrhythmias [30], little research has been conducted in low level isopropyl alcohol exposure populations [31,32].

We hypothesized that the low level isopropyl alcohol exposure could increase blood pressures, which was potentially harmful to human health. In order to verify this hypothesis, we conducted this cross-sectional study in a low isopropyl alcohol exposure population. Besides, we discussed the interactive effect between isopropyl alcohol exposure and other characteristics such as smoking and drinking and explored possible neurogenic mediating factors which may shed light to further studies on the mechanism caused by isopropyl alcohol exposure.

## Materials and Methods

### Study population

Participants were selected from a cohort in Zhongshan, Guangdong province. It was set up by professor He Y to prospectively study the occupational risk factors related cardiovascular diseases in 2012. We conducted this survey in low level isopropyl alcohol exposed workers in one of sub-cohorts from this project in June 2013. The factory from this sub-cohort mainly Produces Information and Communication Technology (ICT) products, including mobile and handheld devices, servers and storage, information equipment, LCD TVs and LCD monitors, network services and communications products. All of the participants were on-the-job. The isopropyl alcohol-exposed workers mainly engaged in maintaining and cleaning of above materials or equipments. The work was performed under local ventilation and characterized by indirect and discontinuous exposure to isopropyl alcohol. Controls were selected from workshop of the same cohort and they did not expose to isopropyl alcohol or other obvious occupational risk factors. They were composed of security guards, electricians and room cleaners. Both groups ruled out the people who had hematological, cardiovascular, respiratory or infectious related diseases. Finally, 200 isopropyl alcohol occupational exposed workers and 83 controls were recruited in this study.

The present study was approved by the Human Subjects Committees of Sun Yat-sen University and all participants provided their written informed consents.

### The exposure assessment

The Zhongshan CDC monitored the air isopropyl alcohol level in different workshops each year according to “Deternination of alcohols in the air of workplace” (GBZ/T160.48–2007). We evaluated last 4 years data at 4 different check points, which would allow accurate assessment. In order to assess internal exposure levels we measured the individual acetone content in urine by reference to “headspace-gas chromatography examination methods for ethanol, methanol, n-propanol, aldehyde, acetone, isopropanol and n-butanol in blood and urine” (GA/T 1073–2013).

### Data collection

We used General Health Questionnaire to collect the basic information of all participants. The items mainly include age, gender, educational level, marital status, personal income, working years, diseases history, family history, smoking status and drinking status and so on. Height, weight, chest circumference, waist and hip circumference were also collected by specially trained interviewers.

Healthy physical examination and blood collection was performed by professional physicians under standard operating procedures. We used X-ray, type-B ultrasonic to exclude possible organic lesions. Total serum cholesterol(TCHO), low density lipoprotein cholesterol (LDL-c), triglycerides(TG) and creatinine(CR) were detected by automatic biochemical analyzer(Hitachi, Japan).

The blood pressure was measured by physicians with a standard mercury sphygmomanometer with a 14-cm cuff. The mean blood pressure of the right and left arm was used as each participant’s final artery systolic blood pressure (SBP) and diastolic blood pressure (DBP). Heart rate was recorded as beats per minute. Pulse blood pressure (PBP) was calculated as the mean systolic minus the mean diastolic blood pressure. Mean blood pressure (MBP) was defined as (SBP+2*DBP) /3. Lastly, we used reversed-phase HPLC fluorescence method(Shimadzu corporation, Kyoto, Japan) to determine the metabolites of MNTs in the urine of the participants. These metabolites were vanillylmandelic acid(VMA), dihydroxyphenylacetic acid(DOPAC), 5-hydroxy indole acetic acid(5-HIAA) and homovanillic acid (HVA) as described previously [33].

### Statistical analysis

We employed Epidata3.1 software to set up database and entry the data. The data was analyzed using the SPSS17.0 software package(SPSSInc., Chicago, IL, USA). All of the *p* values were two-sided and statistical significance was assessed at a value less than 0.05. The values were expressed in the form of mean±SD(standard deviation) for the continuous indexes or the percent with number of cases for the categorical indexes. The independent sample T-test or chi-square test was used to compare the differences of basic characteristics between two groups.

Linear regression models were used to analyze the data. Firstly, we conducted a step by step multiple linear regression analysis to obtain the significant covariates and to estimate the crude effect of the exposure to blood pressures. Then, an entered multiple linear regression models were used to test the association between isopropyl alcohol exposure and blood pressures and to analyze the interaction effects of isopropyl alcohol exposure with characteristics such as smoking and drinking on arterial blood pressures with adjusting for potential covariates. The cross-product term for testing isopropyl alcohol exposure and characteristics interactions were expressed as isopropyl alcohol *characteristic (i.e., isopropyl alcohol*smoking). We tested interactions between isopropyl alcohol exposure and each of the characteristics of interest regardless of the main effects of isopropyl alcohol exposure or characteristics themselves.

The mediating effects of the metabolites on the association between isopropyl alcohol exposure and blood pressures were determined with a series of linear regression models with adjusting for potential covariates. According to Baron and Kenny [34], mediation is demonstrated when the main independent variable (i.e., isopropyl alcohol exposure) is significantly associated with the main dependent variable (i.e., MBP); the independent variable (i.e., isopropyl alcohol exposure) is significantly associated with the mediator variable (i.e.,5-HIAA); and the mediator variable (i.e., 5-HIAA) is significantly associated with the dependent variable (i.e., MBP) when the independent variable (i.e., isopropyl alcohol exposure) is controlled for. The size of the mediation effect was calculated by *ab/(ab+c’)* [35], where *a* is the coefficient relating the independent variable to the mediator, *b* is the coefficient relating the mediator to the dependent variable while adjusting for the independent variable, and *c’* is the coefficient relating the independent variable to the dependent variable while adjusting for the mediator.

## Results

### The basic characteristics of the subjects

The study eventually collected 283 qualified samples (exposed 200, controls 83). There were 234 males and 49 females. The majority of all participants were young with the age range from 18 to 42 years old. The mean age of exposed group was (24.5±3.6) years and the control was (22.5±5.0) years. The isopropyl alcohol exposure years were (2.5±2.2). As expected, variables such as gender, nationality, birth place, marital status, income, smoking history, family history of CVDs in exposed group were comparable with controls. While the differences of body mass index (BMI), drinking, LDL-c and TCHO levels between the exposed group and controls were statistical significance (*p*<0.05) (Table 1). Value assignments and missing data in participants were shown in S1 Table.

**Table 1:** Basic characteristics among workers exposed to isopropyl alcohol and controls (N = 283).

Characteristic	Exposed(n = 200)	Controls(n = 83)	P
**Sex, %(N)**			0.390^#^
**Male**	84.0(168)	79.5(66)	
**Female**	16.0(32)	20.5(17)	
**Age(years), mean ±SD**	24.5±3.6	22.5±5.0	0.001*
**BMI(kg/m2), mean ±SD**	21.2±2.9	20.0±2.3	<0.001*
**Waist circumstance(cm), mean ±SD**	76.5±9.7	72.3±7.9	<0.001*
**Race, %(N)**			0.257^#^
**Han**	92.3(180)	88.0(73)	
**Others**	7.7(15)	12.0(10)	
**Birth place, %(N)**			0.157^#^
**Town**	32.8(64)	24.1(20)	
**Village**	67.2(131)	75.9(63)	
**Highest school level, %(N)**			0.028^#^
**Primary**	1.0(2)	0.0(0)	
**Technical**	72.2(142)	86.7(72)	
**College or above**	26.8(53)	13.3(11)	
**Marital status, %(N)**			0.260^#^
**married**	28.6(57)	21.7(18)	
**Unmarried**	71.4(140)	75.9(63)	
**Others**	1.0(2)	2.4(2)	
**Income(¥), %(N)**			0.131^#^
**≤2000**	28.9(57)	40.5(32)	
**≤4000**	50.3(99)	44.3(35)	
**≤6000**	13.7(27)	6.3(5)	
**>6000**	7.1(14)	8.9(7)	
**Smoking history, %(N)**			0.779^#^
**Yes**	33.3(61)	31.3(26)	
**No**	66.7(122)	68.7(57)	
**Smoking index(pack-year), mean ±SD**	1.5 ±1.45	1.0 ±1.5	0.134*
**Drinking history,%(N)**			0.002^#^
**Yes**	19.6(37)	4.9(4)	
**No**	80.4(152)	95.1(78)	
**Family history of CVDs,%(N)**			0.296^#^
**Yes**	19.4(36)	13.4(11)	
**No**	80.6(150)	86.6(71)	
**isopropyl alcohol contact(years), mean ±SD**	2.5±2.2	-	-
**Working time(hours), mean ±SD**	9.8±5.4	9.7±1.2	0.953*
**LDL-C(ug/g CR), mean ±SD**	36.9±45.8	28.4±11.3	0.029*
**TG(ug/g CR), mean ±SD**	22.2±19.6	13.8±7.3	<0.001*
**TCHO(ug/g CR), mean ±SD**	98.6±11.2	58.1±15.1	<0.001*
**Urinary acetone(mg/L), mean ±SD**	3.0±5.3	1.4±0.8	0.005*

*p: value of independent sample T-test.

#*p*: value of χ² test. CVDs: cardiovascular diseases, TG: Triglycerides, TCHO: Total cholesterol, CR: creatinine.

### Isopropyl alcohol exposure level

The concentrations of air isopropyl alcohol in all monitored workshops and time points were less than 100mg/m^3^ (S2 Table). Thus, we can regard this contact level as a low level exposure comparing to the occupational exposure limit (700mg/m^3^) (GBZ2.1–2007). Urinary acetone in exposed group was (3.0±5.3)mg/L, higher than that of control group (1.4±0.8) mg/L, the difference was significant (*p*< 0.05) (Table 1). Other organic components in the workplace, including benzene, acetone, xylene, toluene and methanol were not detectable (not mentioned).

### Low level of isopropyl alcohol exposure was associated with arterial blood pressures

BMI and birth place were potential covariates for SBP; age, race, waist circumstance and drinking were potential covariates for DBP; age, waist circumstance and birth place were potential covariates for MBP; age and BMI were potential covariates for PBP (S3 Table). Notably, SBP and DBP were 5 mmHg higher than that of controls, and MBP was increased 4 mmHg in exposed group. Linear regression results showed that SBP, DBP and MBP were obviously associated with isopropyl alcohol exposure, the associations remained significant after adjusting for potential covariates (*p*<0.05). However, there was no difference when it came to PBP (*p*>0.05) (Table 2). In addition, we analyzed exposure-response relationship between isopropyl alcohol exposure and blood pressures but we did not observe a dose-response relationship under this exposure level (S4 Table).

**Table 2:** Blood pressures (mmHg) in isopropyl alcohol exposed workers and controls.

Blood pressure	Exposed	Controls	D-value	P_crude_	P_ad._
**SBP, mean ±SD**	123±10	118±11	5	0.003	0.012
**DBP, mean ±SD**	79±7	74±7	5	0.001	0.005
**MBP, mean ±SD**	93±8	89±9	4	0.001	0.008
**PBP, mean ±SD**	44±7	44±8	0	0.725	0.962

SD: standard deviation.SBP: Systolic Blood Pressure. DBP: diastolic blood pressure. MBP: mean blood pressure. PBP: pulse blood pressure. D-value: the mean difference between exposed and controls. *p*_crude_: unadjusted for covariates. *p*_ad._: adjusted for potential covariates.

### The interactions between low level isopropyl alcohol exposure and basic characteristics

**Fig 1:**
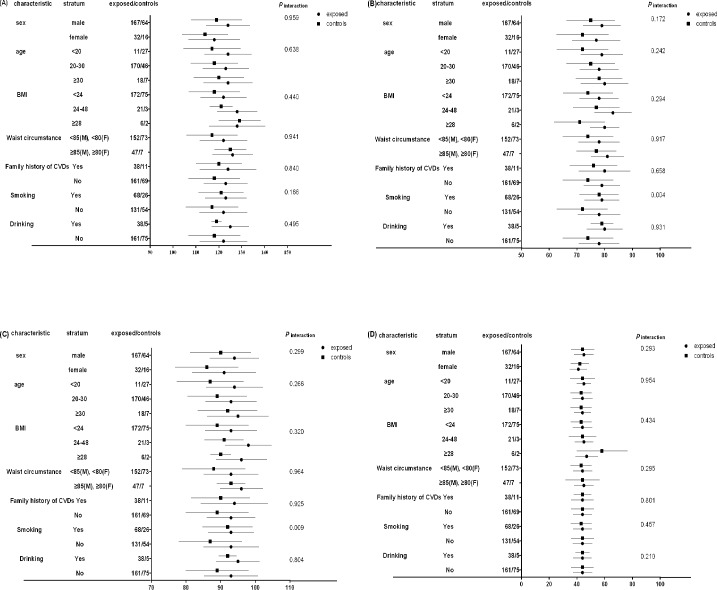
The interactive effects between low level isopropyl alcohol exposure and basic characteristics on arterial blood pressures(mmHg). (A) SBP; (B) DBP; (C) MBP; (D) PBP. SBP: systolic blood pressure. DBP: diastolic blood pressure. MBP: mean blood pressure. PBP: pulse blood pressure.

As shown in Fig 1, participants in exposed group had higher SBP, DBP and MBP than controls in each selected characteristics. Moreover, mean SBP in all exposed sub-layers except for female group were greater than 120mmHg. Interestingly, both DBP and MBP in exposed group were 6mmHg higher than controls among participants who did not smoke whereas when it came to participants who did smoke, the increases were only 1mmHg for DBP and MBP. As indicated, significant interactions were observed between isopropyl alcohol exposure and smoking on DBP and MBP after adjusting for covariates (*p*_interaction_<0.05). We detected no interactions between isopropyl alcohol exposure and sex, age, BMI, waist circumstance, family history of CVDs and drinking (*p*_interaction_>0.1).

### The mediation effect of 5-HIAA on the association between isopropyl alcohol exposure and blood pressures

After adjusting for the potential confounding variables, the exposed group had significantly higher levels of VMA, DOPAC and 5-HIAA than controls (*p*<0.05) (S5 Table). Table 3 presents the results of the correlation between them. It shows that 5-HIAA is significantly and negatively associated with DBP and MBP (*p*<0.05).

**Table 3:** The associations between metabolites of monoamine neural transmitters and arterial blood pressures.

	SBP			DBP			MBP			PBP		
models	Β	S.E.	p	β	S.E.	p	β	S.E.	p	β	S.E.	P
**VMA**	-0.115	0.086	0.183	-0.047	0.064	0.462	-0.071	0.066	0.286	-0.061	0.062	0.324
**DOPAC**	-0.036	0.031	0.240	-0.011	0.023	0.642	-0.023	0.024	0.341	-0.031	0.022	0.164
**5-HIAA**	-0.204	0.139	0.143	-0.206	0.104	0.049	-0.220	0.107	0.040	-0.035	0.101	0.733
**HVA**	-0.080	0.136	0.560	-0.051	0.102	0.620	-0.063	0.104	0.544	-0.023	0.098	0.811

S.E: standard error.SBP: Systolic Blood Pressure. DBP: diastolic blood pressure. MBP: mean arterial pressure. PBP: pulse pressure. VMA: vanillylmandelic acid. DOPAC: dihydroxyphenylacetic acid. 5-HIAA: 5-hydroxy indole acetic acid. HVA: homovanillic acid. P value: adjusted for potential covariates.

**Fig 2:**
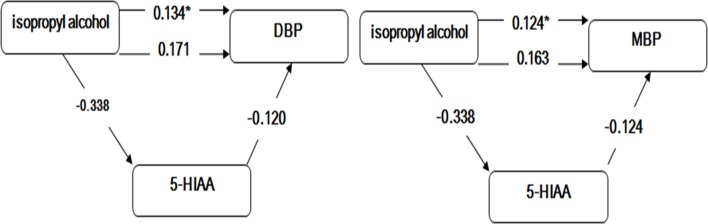
The mediating effects of 5-HIAA on DBP and MBP. *: the standard coefficients relating the independent variable to the dependent variable after adjusting for 5-HIAA and potential covariates with *p*<0.05. DBP: diastolic blood pressure. MBP: mean blood pressure. 5-HIAA: 5-hydroxy indole acetic acid.

Fig 2 illustrates the mediation effect and shows that isopropyl alcohol exposure is significantly associated with DBP(c = 0.171, *p*<0.05) and MBP(c = 0.163, *p*<0.05) after adjusting for the potential covariates. While adjusting for 5-HIAA besides the potential covariates, isopropyl alcohol exposure is remain significantly associated with DBP(c’ = 0.134,*p*<0.05) and MBP (c = 0.124,*p*<0.05); isopropyl alcohol exposure is significantly associated with 5-HIAA(a = -0.338, *p*<0.05); and 5-HIAA is significantly associated with DBP(b = -0.120,*p*<0.05) and MBP(b = -0.124,*p*<0.05) when controlled for isopropyl alcohol exposure. 5-HIAA mediated the association between isopropyl alcohol exposure and DBP, MBP. The meditative effects on DBP and MBP can be expressed as (-0.338 × -0.120)/ [(-0.338 × -0.120) + 0.134] = 23.1% and (-0.338 × -0.124)/ [(-0.338 × -0.124) + 0.124] = 25.3%, respectively.

## Discussion

Cardiovascular diseases are important world’s public health problems [36–38]. It has become increasingly clear that high blood pressure is associated with a variety of cardiovascular and renal events (fatal and nonfatal), including myocardial infarction, stroke, atherosclerosis, aortic aneurysm, hypertensive heart disease, heart failure, peripheral artery disease, and end-stage renal disease. Blood pressure(SBP>110-115mmHg) is now the leading determinant of morbidity and mortality worldwide, responsible for an even greater burden of disease than that conferred by smoking [39]. Given this background, the factors influencing blood pressure will become key area of concern for researchers, including environmental and occupational factors [40,41].

To our knowledge, this study provided first evidence that low level isopropyl alcohol exposure was associated with increased blood pressure. Previous studies found that high concentrations of isopropyl alcohol can reduce blood pressure, leading to hypotension in isopropyl alcohol poisoning population. Here we found that low level isopropyl alcohol could increase arterial blood pressures. This phenomenon may share a special effect called “hormesis” which can be observed in many chemicals at low level exposure [42,43].In addition, we observed an interaction between isopropyl alcohol exposure and smoking and discovered a mediating role of 5-HIAA on low level isopropyl alcohol exposure induced increase of blood pressures, which might shed light for further mechanism studies.

Firstly, our exposure assessment showed that the exposed subjects had a low level of isopropyl alcohol exposure. On the one hand, we evaluated isopropyl alcohol concentration in the air of working environment by reviewing the monitored data in last 4 years. On the other hand, we detected the individual internal exposure level through urine. Both of the results were far less than the exposure limit. But they are greater than that in controls. Other organic components in the workplace, including benzene, acetone, xylene, toluene and methanol were not detectable (not mentioned), which suggesting that isopropyl alcohol was the main occupational risk factor. Few studies were focused on the health effect of the low level isopropyl alcohol exposure.

Secondly, in this study, we demonstrated that low level isopropyl alcohol exposure was associated with elevated blood pressures even after adjusted for covariates. Although among the basic characteristics we observed that age and educational level were different between the two groups. As can be seen from the results, most of the participants were young people and the difference was less than 3 years as the mean age of exposed group was 24.5 (SD = 3.6) years and the control was 22.5 (SD = 5.0) years. To avoid confounding, we compared the blood pressures after stratified by age. It showed that both SBP and DBP were still higher in exposed group than that in controls. As for educational level, the percentage of high educational level in exposed group was higher than that in controls so that it may weaken the effects of low level isopropyl alcohol exposure on SBP and DBP due to its protection effect on blood pressure. Finally, we found low level isopropyl alcohol exposure was associated with elevated blood pressures even after adjusted for above bias or other covariates by regression models. Similarly, both SBP and DBP were increased, which indicated that arterial blood pressures could be activated by low level isopropyl alcohol exposure. We analyzed the relationship between the different exposure levels and blood pressure according to the concentration of acetone in urine. Although it suggested an increased tendency, we have not provided formal proof yet that there is a dose—response relationship between them. This may be related to the narrow range of exposure level itself. Another possible reason is that other individual difference such as genetic factors, behavior and life style interact with low level isopropyl alcohol exposure, leading to the modified results. Above all, these results suggest that even low level isopropyl alcohol exposure could affect blood pressure. It was of vital importance to pay attention to the change of blood pressure in workers who had isopropyl alcohol exposure.

Thirdly, our focus shifts to further potential mechanisms studies, based on the activated effects of low level isopropyl alcohol exposure on blood pressures showed above. The results proved that smoking could interact with isopropyl alcohol exposure. Smoking is an important traditional risk factor for cardiovascular disease and smoking can interact with many factors [44, 45]. Our research showed that after sub-layered by smoking, the blood pressure in isopropyl alcohol exposure group was higher than that of controls at each sub-layer. And it was interesting to note that SBP, DBP and MBP in exposed group were 5mmHg, 6mmHg and 6mmHg higher than controls in participants who did not smoke, whereas the increases were only 2mmHg, 1mmHgand 1mmHg for SBP, DBP and MBP in smoking participants, suggesting that there probably be an interaction between isopropyl alcohol exposure and smoking. Thus, we employed the linear regression models to explore the interaction of the two factors. According to the results,the interactive effect was stronger than each of the main effect, but weaker than the sum of the two. That was, low level isopropyl alcohol exposure and smoking had a negative interaction. Thus, further mechanism studies are needed to clarify their interactive effect from the cellular and molecular levels. We did not observe an interaction between low level isopropyl alcohol exposure and sex, age, BMI, waist circumstance, family history of CVDs or drinking. For drinking, we think further evidences are needed to reach the conclusions because our study was limited by the few sample size and little alcohol drinking among the participants. In general, the above results suggest that isopropyl alcohol exposure could interact with smoking to affect blood pressure.

Last but not least, through the mediating effect analysis, our study further validated the mediator role of 5-HIAA between low level isopropyl alcohol exposure and elevated blood pressure. The results demonstrated that 5-HIAA played a mediation role in the association between isopropyl alcohol exposure and blood pressures. As we all know, MNTs (including adrenaline, norepinephrine, dopamine and serotonin) are the earliest discovered central neurotransmitter, which is closely related to human health and diseases including hypertension [46]. And on this basis we further studied the associations between arterial blood pressures and the metabolites of these transmitters. The neural effects of serotonin (5-Hydroxytryptamine, 5-HT) have been well investigated and understood and it is well established that the serotonergic system (SS) plays important roles in the pathogenesis of cardiovascular diseases [47,48]. The role played by 5-HT in hypertension is still unclear. In the human, free circulating 5-HT is elevated, and the uptake of 5-HT appears to be impaired in the platelet of the hypertensive human such that the platelet appears “activated” [49,50]. By contrast, in pregnancy-induced hypertension, platelet content of 5-HT is increased and release of 5-HT is reduced [51] or shown to be unchanged [52]. Filshie et al. [53] found that urinary5-HIAA was not elevated in women with pregnancy induced hypertension compared with women with normal pregnancy. Our results showed that DBP and MBP were significantly and negatively associated with urinary5-HIAA, which is the main metabolites of 5-HT. The data illustrate that the activated effect of isopropyl alcohol on blood pressure be partly regulated by SS. It is of vital importance for researchers and administrative departments to pay more attention to the toxicity caused by isopropyl alcohol exposure.

## Conclusions

From the discussion, one may conclude that low level isopropyl alcohol exposure was associated with increased blood pressure. Besides, our work supported the view of chemical hormesis and tested our hypothesis of its effect on blood pressures. We demonstrated that the interaction effect of isopropyl alcohol and smoking on blood pressure and found the mediation effect of 5-HIAA on the association between isopropyl alcohol exposure and blood pressures based on an occupational population study. More studies are needed to fully study the cardiovascular toxicity and mechanism of isopropyl alcohol and its interaction with other risk factors.

## Supporting Information

S1 File The General Health Questionnaire (Chinese and English version). (PDF)

S2 File Completed STROBE_checklist. (DOC)

S1 Table Value assignments and missing data in this study. (DOC)

S2 Table Isopropyl alcohol concentration (mg/m3) in the air of workshop. (DOC)

S3 Table Potential covariates of arterial blood pressures among basiccharacteris-tics. (DOC)

S4 Table Exposure-response relationship between urinary acetone concentrations among workers exposed to low level isopropyl alcohol and controls, and arterial blood pressures(mmHg)*. (DOC)

S5 Table The metabolites of monoamine neural transmitters in isopropyl alcohol exposed workers and controls. (DOC)

## References

[1] O’Neil MJ, ed. The Merck Index: An Encyclopedia of Chemicals, Drugs and Biologicals. 15th ed. London: Royal Society of Chemistry; 2013.

[2] Mowry JB, Spyker DA, Cantilena LR Jr, Bailey JE, Ford M. 2012 Annual Report of the American Association of Poison Control Centers’ National Poison Data System (NPDS): 30th Annual Report. Clin Toxicol (Phila) 2013; 51: 949–1229. 24359283 10.3109/15563650.2013.863906

[3] Lewis RJ. Sax’s Dangerous Properties of Industrial Materials,Vol 1–5 12th ed. New York: John Wiley & Sons; 2012.

[4] Martz W. A lethal ingestion of a household cleaner containing pine oil and isopropanol. J Anal Toxicol, 2010, 34(1):49–52. 20109303 10.1093/jat/34.1.49

[5] Nwosu ME, Golomb MR. Cerebral sinovenous thrombosis associated with isopropanol ingestion in an infant. J Child Neurol, 2009, 24(3):349–353. 10.1177/0883073808322664 19258296

[6] Stremski E, Hennes H. Accidental isopropanol ingestion in children. Pediatr Emerg Care, 2000, 16(4):238–240. 10966340 10.1097/00006565-200008000-00005

[7] Vicas IM, Beck R. Fatal inhalational isopropyl alcohol poisoning in a neonate. J Toxicol Clin Toxicol, 1993, 31(3):473–481. 8355323 10.3109/15563659309000415

[8] Jones AW. Elimination half-life of acetone in humans: case reports and review of the literature. J Anal Toxicol, 2000, 24(1):8–10. 10654562 10.1093/jat/24.1.8

[9] Martinez TT, Jaeger RW, deCastro FJ, Thompson MW, Hamilton MF. A comparison of the absorption and metabolism of isopropyl alcohol by oral, dermal and inhalation routes. Vet Hum Toxicol, 1986, 28(3):233–236. 3727356

[10] Wood JN, Carney J, Szczepanski K, Calello DP, Hurt H. Transplacental isopropanol exposure: case report and review of metabolic principles. J Perinatol, 2007, 27(3):183–185. 17314988 10.1038/sj.jp.7211646

[11] Daniel DR, Mcanalley BH, Garriott JC. Isopropyl alcohol metabolism after acute intoxication in humans. J Anal Toxicol, 1981, 5(3):110–112. 7265921 10.1093/jat/5.3.110

[12] Nordmann R, Ribiere C, Rouach H, Beauge F, Giudicelli Y, Nordmann J. Metabolic pathways involved in the oxidation of isopropanol into acetone by the intact rat. Life Sci,1973, 13(7):919–932. 4358273 10.1016/0024-3205(73)90082-9

[13] Natowicz M, Donahue J, Gorman L, Kane M, McKissick J, Shaw L. Pharmacokinetic analysis of a case of isopropanol intoxication. Clin Chem,1985,31(2):326–328. 3967375

[14] Dalziel K, Dickinson FM. The kinetics and mechanism of liver alcohol dehydrogenase with primary and secondary alcohols as substrates. Biochem J, 1966, 100(1):34–46. 4290533 10.1042/bj1000034 PMC1265089

[15] Leeper SC, Almatari AL, Ingram JD, Ferslew KE. Topical absorption of isopropyl alcohol induced cardiac and neurologic deficits in an adult female with intact skin. Vet Hum Toxicol, 2000, 42(1):15–17. 10670080

[16] Slaughter RJ1, Mason RW, Beasley DM, Vale JA, Schep LJ. Isopropanol poisoning. Clin Toxicol (Phila), 2014, 52(5):470–478. 24815348 10.3109/15563650.2014.914527

[17] Sethre T, Läubli T, Berode M, Krueger H. Neurobehavioural effects of experimental isopropanol exposure. Int Arch Occup Environ Health, 2000, 73(2):105–112. 10741508 10.1007/s004200050015

[18] Mueller-Kronast N, Rabinstein AA, Voung L, Forteza AM. Isopropanol intoxication mimicking basilar artery thrombosis. Neurology, 2003, 61(10):1456–1457. 14638985 10.1212/01.wnl.0000094205.26168.43

[19] Nwosu ME, Golomb MR. Cerebral sinovenous thrombosis associated with isopropanol ingestion in an infant. J Child Neurol, 2009, 24(3):349–353. 10.1177/0883073808322664 19258296

[20] Faber WD, Pavkov KL, Gingell R. Review of reproductive and developmental toxicity studies with isopropanol. Birth Defects Res B Dev Reprod Toxicol, 2008, 83(5):459–476. 10.1002/bdrb.20167 18924148

[21] Allen B1, Gentry R, Shipp A, Van Landingham C. Calculation of benchmark doses for reproductive and developmental toxicity observed after exposure to isopropanol. Regul Toxicol Pharmacol, 1998, 28(1):38–44. 9784431 10.1006/rtph.1998.1226

[22] Palermo AM, Mudry MD. Genotoxic damage induced by isopropanol in germinal and somatic cells of Drosophila melanogaster. Mutat Res, 2011, 726(2):215–221. 10.1016/j.mrgentox.2011.09.016 22001194

[23] Nelson BK, Brightwell WS, MacKenzie-Taylor DR, Khan A, Burg JR, Weigel WW, et al Teratogenicity of n-propanol and isopropanol administered at high inhalation concentrations to rats. Food Chem Toxicol, 1988, 26(3):247–254. 3366425 10.1016/0278-6915(88)90126-3

[24] Désy O, Carignan D, Caruso M, de Campos-Lima PO. Immunosuppressive effect of isopropanol: down-regulation of cytokine production results from the alteration of discrete transcriptional pathways in activated lymphocytes. J Immunol, 2008, 181(4):2348–2355. 18684924 10.4049/jimmunol.181.4.2348

[25] Carignan D, Desy O, de Campos-Lima PO. The dysregulation of the monocyte/macrophage effector function induced by isopropanol is mediated by the defective activation of distinct members of the AP-1 family of transcription factors. Toxicol Sci, 2012, 125(1):144–156. 10.1093/toxsci/kfr283 22020770

[26] Freireich AW, Cinque TJ, Xanthaky G, Landau D. Hemodialysis for isopropanol poisoning. N Engl J Med, 1967, 277(13):699–700. 6039883 10.1056/NEJM196709282771308

[27] Clark J D. Isopropyl alcohol intoxication. J Emerg Nurs, 2010, 36(1):81–82. 10.1016/j.jen.2009.10.006 20109790

[28] Emadi A, Coberly L. Intoxication of a hospitalized patient with an isopropanol-based hand sanitizer. N Engl J Med, 2007, 356(5):530–531. 17267921 10.1056/NEJMc063237

[29] Adelson L. Fatal intoxication with isopropyl alcohol (rubbing alcohol). Am J Clin Pathol, 1962, 38:144–151. 13859391 10.1093/ajcp/38.2.144

[30] Peng LW, Janga R, Lien YH. Isopropyl alcohol-induced pseudo-azotemia: taking advantage of a laboratory error. Am J Med, 2006, 119(8):e9 16887402 10.1016/j.amjmed.2005.12.024

[31] Iavicoli I, Fontana L, Iavicoli S. Modifications of hepatic transaminases in workers exposed to low doses of isopropanol. G Ital Med Lav Ergon, 2007, 29(3 Suppl):271–272. 18409681

[32] Sethre T, Läubli T, Hangartner M, Berode M, Krueger H. Isopropanol and methylformate exposure in a foundry: exposure data and neurobehavioural measurements. Int Arch Occup Environ Health, 2000, 73(8):528–536. 11100947 10.1007/s004200000178

[33] Jiang K, Wu H, Qin W, Gu G, Yu S. Determination of four biogenic amine metabolites in urine by high-performance liquid chromatography. Zhonghua Lao Dong Wei Sheng Zhi Ye Bing Za Zhi, 2014, 32(2):140–142. 24630022

[34] Baron RM, Kenny DA. The moderator-mediator variable distinction in social psychological research: conceptual, strategic, and statistical considerations. J Pers Soc Psychol, 1986, 51(6):1173–1182. 3806354 10.1037//0022-3514.51.6.1173

[35] Mackinnon DP, Fairchild AJ, Fritz MS. Mediation analysis. Annu Rev Psychol, 2007, 58:593–614. 16968208 10.1146/annurev.psych.58.110405.085542 PMC2819368

[36] Zahra A, Lee EW, Sun LY, Park JH. Cardiovascular disease and diabetes mortality, and their relation to socio-economical, environmental, and health behavioural factors in worldwide view. Public Health, 2015, 129(4):385–395. 10.1016/j.puhe.2015.01.013 25724438

[37] Nascimento BR, Brant LC, Moraes DN, Ribeiro AL. Global health and cardiovascular disease. Heart, 2014, 100(22):1743–1749. 10.1136/heartjnl-2014-306026 25327515

[38] Cosselman KE, Navas-Acien A, Kaufman JD. Environmental factors in cardiovascular disease. Nat Rev Cardiol, 2015, 12(11):627–642. 10.1038/nrcardio.2015.152 26461967

[39] Lim SS, Vos T, Flaxman AD, Danaei G, Shibuya K, Adair-Rohani H, et al A comparative risk assessment of burden of disease and injury attributable to 67 risk factors and risk factor clusters in 21 regions, 1990–2010: a systematic analysis for the Global Burden of Disease Study 2010. Lancet, 2012, 380(9859):2224–2260. 10.1016/S0140-6736(12)61766-8 23245609 PMC4156511

[40] Leow MK. Environmental origins of hypertension: phylogeny, ontogeny and epigenetics. Hypertens Res, 2015, 38(5):299–307. 10.1038/hr.2015.7 25693856

[41] Rapisarda V, Ledda C, Ferrante M, Fiore M, Cocuzza S, Bracci M, Fenga C. Blood pressure and occupational exposure to noise and lead (Pb): A cross-sectional study. Toxicol Ind Health,2015, 10.1177/0748233715576616 [Epub ahead of print] 25883097

[42] Calabrese EJ, Baldwin LA. Hormesis: the dose-response revolution. Annu Rev Pharmacol Toxicol, 2003, 43:175–197. 12195028 10.1146/annurev.pharmtox.43.100901.140223

[43] Calabrese EJ, Blain RB. The hormesis database: the occurrence of hormetic dose responses in the toxicological literature. Regul Toxicol Pharmacol, 2011, 61(1):73–81. 10.1016/j.yrtph.2011.06.003 21699952

[44] Messner B, Bernhard D. Smoking and cardiovascular disease: mechanisms of endothelial dysfunction and early atherogenesis. Arterioscler Thromb Vasc Biol, 2014, 34(3):509–515. 10.1161/ATVBAHA.113.300156 24554606

[45] Grammer TB, Hoffmann MM, Scharnagl H, Kleber ME, Silbernagel G, Pilz S, et al Smoking, apolipoprotein E genotypes, and mortality (the Ludwigshafen RIsk and Cardiovascular Health study). Eur Heart J, 2013, 34(17):1298–1305. 10.1093/eurheartj/eht001 23382465

[46] Hyland K. Clinical utility of monoamine neurotransmitter metabolite analysis in cerebrospinal fluid. Clin Chem, 2008, 54(4):633–641. 10.1373/clinchem.2007.099986 18310141

[47] Vikenes K, Farstad M, Nordrehaug J E. Serotonin is associated with coronary artery disease and cardiac events. Circulation, 1999, 100(5):483–489. 10430761 10.1161/01.cir.100.5.483

[48] Côté F, Fligny C, Fromes Y, Mallet J, Vodjdani G. Recent advances in understanding serotonin regulation of cardiovascular function. Trends Mol Med, 2004, 10(5):232–238. 15121050 10.1016/j.molmed.2004.03.007

[49] Persson B, Gradin K, Pettersson A, Hedner T. Antihypertensive effects ofketanserin and ritanserin in the spontaneously hypertensive rat. J CardiovascPharmacol, 1988, 11(S1):S22–S24. 2459509

[50] Carrasco G, Cruz MA, Gallardo V, Miguel P, Lagos M, González C. Plasma and platelet concentration and platelet uptake of serotonin in normal and pre-eclamptic pregnancies. Life Sci, 1998, 62(15):1323–1332. 9566774 10.1016/s0024-3205(98)00066-6

[51] Gujrati VR, Goyal A, Gaur SP, Singh N, Shanker K, Chandravati. Relevance of platelet serotonergic mechanisms in pregnancy induced hypertension. Life Sci, 1994, 55(4):327–335. 8028450 10.1016/0024-3205(94)00735-7

[52] Jelen I, Fananapazir L, Crawford T B. The possible relation between late pregnancy hypertension and 5-hydroxytryptamine levels in maternal blood. Br J Obstet Gynaecol, 1979, 86(6):468–471. 465398 10.1111/j.1471-0528.1979.tb10791.x

[53] Filshie GM, Maynard P, Hutter C, Cooper JC, Robinson G, Rubin P. Urinary 5-hydroxyindole acetate concentration in pregnancy induced hypertension. BMJ, 1992, 304(6836):1223 1381251 10.1136/bmj.304.6836.1223 PMC1881811

