# Supplementary material for: The Activation Effects of Low Level Isopropyl Alcohol Exposure on Arterial Blood Pressures Are Associated with Decreased 5-Hydroxyindole Acetic Acid in Urine
Source: PLoS One. 2016 Sep 13;11(9):e0162762. doi: 10.1371/journal.pone.0162762 (PMC5021351; doi:10.1371/journal.pone.0162762)
Supplement: S1 File — (PDF) [file pone.0162762.s001.pdf]

职工健康调查问卷

调查日期：20_____ 年_______月 _______日　　　　　　　编号：

您好：

我是中山大学公共卫生学院的调查员，我们拟开展职业人群健康状况的调查，内容包括您过去的健康状况、职业史、饮食和其他生活习惯等。全部调查资料是保密的，您的姓名和其它可以识别您的资料不会出现在任何调查报告上。

知情同意书

本人已了解这次健康调查的描述，并对调查员的介绍感到满意，我自愿参加这项询问调查，并同意提供我的血尿样本。

调查对象签名：__________

签名日期：_______年____月___日

调查员陈述

我已向调查对象充分解释了有关事项，调查对象对本调查已有足够的了解。

调查员签名：__________

签名日期：_______年____月___日

姓名：____________ 出生日期：___________ 所在车间：__________ 工种：__________

民族：_____；身高：_____cm；体重：_____kg；

腰围：______cm；臀围：______cm；胸围：______cm

1. 性别_____：1=男；　2=女　☐
2. 您生长地是______：1=城市　2=县城　3=乡镇　4=农村　☐
3. 您的教育程度是______：　☐

   0=无正式教育；　1=小学 1-3 年级；　2=小学 5-6 年级；　3=初中；

   4=高中/中专；　5=大专或大学；　6=硕士或以上；
4. 您目前的婚姻状况是：_____　☐

   1=已婚；　2=离婚；　3=丧偶；　4=分居；　5=未婚；　6=其他
5. 您家每月人均收入为（包含实物收入，如农产品）：____________　☐

   1=500 元以下　2=501~1000 元　3=1001~1500 元　4=1501~2000 元

5=2001~3000 元　　6=3001~4000 元　　7=4001~6000 元　　8=6000 元以上

6. 您是否有过规律性吸烟（如连续吸烟 6 个月，每天至少吸 1 支烟）？： □

1=是；　2=否*（选择“否”者直接跳到 11 题）*

7. 您大约是多大年龄开始规律性吸烟（连续 6 个月，>1 支/天）：_______岁 □

8. 在吸烟期间，您平均每天吸____支烟；累计吸烟有多久了：_____年____ 月 □□

9. 您现在是否已经戒烟：1=否；　2=是，那么您戒了多久：_____年____ 月 □□

10. 在您过去的一年中，是否经常有人室内在您身边抽烟（平均每天多过 1 支烟或 5 分钟）？

1=是；　2=否 □

11. 您是否有过规律性饮酒（要连续 6 个月，每周至少饮一次酒）？ □

1=是；　2=否*（选择“否”者直接跳到 15 题）*

12. 您是多大年龄开始规律性饮酒（连续 6 个月，>1 次/周）：________岁 □

13. 在规律喝酒的这些年里，您估计平均每周饮几两酒？

啤酒：______两/周 ；葡萄酒：_____两/周 ；白酒：_______两/周 ； □□□

14. 您现在是否已经戒酒：0=否；　1=是，那么您戒了多长时间：_____年____ 月 □□□

15. 您平常的健康状况是_______：1=良好；　2=患有疾病，疾病名称：_____________ □

16. 您家族是否有人患有下列疾病：（可多选） □

1=糖尿病；　2=高血压；　3=高血脂；　4=冠心病或动脉硬化；　5=中风；　6=癌症；　7=无

父：_______；母：______；祖或外祖父母：______；兄弟姐妹：______；

17. 您所处工作是否接触以下一些特殊的物质或不利的环境:_____（可多选） □□

1=强噪音；　2=高温环境；　3=灰尘或粉尘；　4=放射线；　5=农药、杀虫剂；

6=重金属（如开矿、电镀工、电焊工、电池工人，油漆或涂料工，印染工人等行业）；

18. 详细职业史（不同工种应分别记录）

时间（年、月）	工作地点（市/县）	工种
至		
至		
至		

Staff Health Questionnaire

Survey Date:__________________ serial number:_________

Hello!

We are investigators from Sun Yat-sen University School of Public Health. We intend to investigate general health status of you, including your personal basic information, living habits, medical history and occupational history. All initial survey data will keep secret. We ensure that your name and other information what can identify you do not appear on any report.

INFORMED CONSENT

I have understood this Health Survey, and I am satisfied with the introduction of the investigator. I volunteer for the survey and agree to provide my blood and urine samples for scientific research.

Signature: __________

Data:__________

INVESTIGATORS STATEMENT

I have already fully explained related matters of this survey and the participants have fully understood the survey.

Signature: __________

Data:__________

Name: ____________ Birthday: ______ Workshop: __________ Type of work:__________

Ethnic group: _____;

Height: _____cm; Weight: _____kg;

Waist: ______cm; Hip circumference: ______cm; Chest circumference: ______cm

1. Gender _____:1=male; 2=female ☐
2. Your birth place______:1=town 2=village ☐
3. Your highest school level______: ☐

   1= primary; 2= technical; 3= College or above;
4. Your current marital status:_____ ☐

1=married; 2=unmarried; 3=others;

5. Your home average income per month:____________ □

1=under 2000RMB 2=2000~4000RMB 3=4001~6000RMB 4=above 6000RMB

6. Have you ever regular smoking (continuous smoking for six months, smoking at least 1 cigarette per day)？: □

1=yes; 2=no***(If select "no", skip to question 11)***

7. How old are you when you start regular smoking: _______years old □

8. During smoking, your average daily cigarettes are____; How long have you smoked: _____years____ months □□

9. Have you quit smoking:1=no; 2=yes，and how long: _____years __months □□

10. In last year, has anybody often smoking indoors at your side (on average more than one cigarette per day or 5 minutes)? 1 = yes; 2 = no □

11. Have you ever regular drinking (continuous drinking for six months, drinking at least once per day)？: 1=yes; 2=no***(If select "no", skip to question 15)*** □

12. How old are you when you start regular drinking: _______years old □

13. In those years, how much did you drink per week？ □□□

beer: ___ounce/week ;grape wine:__ ounce/week ;white wine:____ ounce/week;

14. Have you gave up drinking:1=no; 2=yes，and how long: _____years__months □□□

15. Your health is usually _______:1=good; 2=have diseases，what kind of disease__ □

16. Does your family suffer from the following diseases:（Multiple choice） □

1=diabetes; 2=hypertension; 3= high cholesterol; 4= coronary artery disease or atherosclerosis; 5=stroke; 6=cancer; 7=none

father: _______;mother:______; parental or maternal grandparents: ______;brothers or sisters:______;

17. whether you are in contact with some of the following specific substances or adverse factors in your working environment?:_____ （Multiple choice） □□

1=noise; 2= high-temperature; 3= dirt or dust; 4= radiation; 5= Pesticides, insecticides;

6=heavy metals

18. Occupational history

Working Date	Workplace	Type of work
From______to______	______________________	______________________
From______to______	______________________	______________________
From______to______	______________________	______________________
