# Supplementary material for: The Activation Effects of Low Level Isopropyl Alcohol Exposure on Arterial Blood Pressures Are Associated with Decreased 5-Hydroxyindole Acetic Acid in Urine
Source: PLoS One. 2016 Sep 13;11(9):e0162762. doi: 10.1371/journal.pone.0162762 (PMC5021351; doi:10.1371/journal.pone.0162762)
Supplement: S2 File — (DOC) [file pone.0162762.s002.doc]

STROBE Statement—checklist of items that should be included in reports of observational studies

|  | Item No. | Recommendation | Page  No. | Relevant text from manuscript |
| --- | --- | --- | --- | --- |
| **Title and abstract** | 1 | (*a*) Indicate the study’s design with a commonly used term in the title or the abstract | 1 | The activation effects of low level isopropyl alcohol exposure on arterial blood pressures are associated with decreased 5-hydroxyindole acetic acid in urine |
|  |  | (*b*) Provide in the abstract an informative and balanced summary of what was done and what was found | 1,2 | Purposes  The objectives of this paper are to study the impact of low level isopropyl alcohol exposure on blood pressure and to explore its potential mechanism.  Methods  This cross-sectional study was based on a prospective occupational cohort in south China, which focusing on occupational risk factors related cardiovascular health problems. A total of 283 participants (200 low isopropyl alcohol exposed workers and 83 controls) was finally enrolled in this study. Linear regression models were used to analyze the relationship between arterial blood pressures and low level isopropyl alcohol exposure. We used mediation method to explore possible mediated roles of neurogenic factors.  Results  Systolic blood pressure (SBP, 123±10 vs. 118±11), diastolic blood pressure (DBP, 79±7 vs. 74±7) and mean blood pressure (MBP, 93±8 vs. 89±9) were different between the exposed group and the control group (p < 0.01). After adjusting for covariates, the difference wasstill significant. Besides, isopropyl alcohol and smoking had an interactive effect on DBP and MBP (*p*< 0.05). Furthermore, we observed a mediated effect of 5-hydroxyindole acetic acid (5-HIAA) on isopropyl alcohol exposure induced arterial blood pressure increase, which accounted for about 25%.  Conclusions  Our results suggest that low level isopropyl alcohol exposure is a potential risk factor for the increased arterial blood pressure and 5-HIAA partly mediates the association between low level isopropyl alcohol exposure and arterial blood pressures. |
| Introduction | | | |  |
| Background/rationale | 2 | Explain the scientific background and rationale for the investigation being reported | 2 | Isopropyl alcohol (isopropanol) is a kind of clear, colorless, flammable organic solvents. It is miscible with water, benzene, chloroform, ethanol or glycerol. Isopropyl alcohol and its raw materials have widely application in pharmacy, coating and printing. Often，isopropyl alcohol can be found in many household detergents, cosmetics, disinfectants and paint thinner. In 2012, there were 9833 Americans exposed to rubbing alcohol products and 4456 were exposed to isopropyl alcohol containing cleaning products or hand sanitizers. Thus, the effects of environmental and occupational isopropyl alcohol exposure become urgently need in-depth studies in future. (…)Although it is clear that high concentration of isopropyl alcohol exposure can lead to hypotension, shockand arrhythmias, little research has been conducted in low level isopropyl alcohol exposure populations. |
| Objectives | 3 | State specific objectives, including any prespecified hypotheses | 2 | We hypothesized that the low level isopropyl alcohol exposure could increase blood pressures, which was potentially harmful to human health. In order to verify this hypothesis, we conducted this cross-sectional study in a low isopropyl alcohol exposure population. In addition, we discussed the interactive effect between isopropyl alcohol exposure and other characteristics such as smoking and drinking and explored possible neurogenic mediating factors which may shed light to further studies on the mechanism caused by isopropyl alcohol exposure. |
| Methods | | | |  |
| Study design | 4 | Present key elements of study design early in the paper | 2 | In order to verify this hypothesis, we conducted this cross-sectional study in a low isopropyl alcohol exposure population. Besides, we discussed the interactive effect between isopropyl alcohol exposure and other characteristics such as smoking and drinking and explored possible neurogenic mediating factors which may shed light to further studies on the mechanism caused by isopropyl alcohol exposure. |
| Setting | 5 | Describe the setting, locations, and relevant dates, including periods of recruitment, exposure, follow-up, and data collection | 3 | Participants were selected from a cohort in Zhongshan, Guangdong province. It was set up by professor He Y to prospectively stuy the occupational risk factors related cardiovascular diseases in 2012.We conducted this survey in low level isopropyl alcohol exposed workers in one of sub-cohorts from this project in June 2013. |
| Participants | 6 | (*a*) *Cohort study*—Give the eligibility criteria, and the sources and methods of selection of participants. Describe methods of follow-up  *Case-control study*—Give the eligibility criteria, and the sources and methods of case ascertainment and control selection. Give the rationale for the choice of cases and controls  *Cross-sectional study*—Give the eligibility criteria, and the sources and methods of selection of participants | 3 | All of the participants were on-the-job. The exposed group mainly engaging in the maintaining and cleaning of above materials or equipments. The work was performed under local ventilation and characterized by indirect and discontinuous exposure to isopropyl alcohol.. Controls were selected from workshop of the same cohort and they did not expose to isopropyl alcohol or other obvious occupational risk factors. They were composed of security guards, electricians and room cleaners. Both groups ruled out these patients who had hematological, cardiovascular, respiratory or infectious related diseases. Finally, 200 isopropyl alcohol occupational exposed workers and 83 controls were recruited in this study. |
|  |  | (*b*)*Cohort study*—For matched studies, give matching criteria and number of exposed and unexposed  *Case-control study*—For matched studies, give matching criteria and the number of controls per case |  |  |
| Variables | 7 | Clearly define all outcomes, exposures, predictors, potential confounders, and effect modifiers. Give diagnostic criteria, if applicable | 3,4 | The items mainly include age, gender, educational level, marital status, personal income, working years, diseases history, family history, smoking status and drinking status and so on. Height, weight, chest circumference, waist and hip circumference were also collected by specially trained interviewers.(…)Total serum cholesterol(TCHO), low density lipoprotein cholesterol (LDL-c), triglycerides(TG) and creatinine(CR) were detected by automatic biochemical analyzer(Hitachi, Japan).(…)The mean blood pressure of the right and left arm was used as each participant’s final artery systolic blood pressure (SBP) and diastolic blood pressure (DBP). Heart rate was recorded as beats per minute. Pulse blood pressure (PBP) was calculated as the mean systolic minus the mean diastolic blood pressure. Mean blood pressure (MBP) was defined as (SBP+2*DBP) /3.(…)These metabolites were vanillylmandelic acid(VMA), dihydroxyphenylacetic acid(DOPAC), 5-hydroxy indole acetic acid(5-HIAA) and homovanillic acid (HVA) as described previously.(…)to obtain the signiﬁcant covariates. |
| Data sources/measurement | 8* | For each variable of interest, give sources of data and details of methods of assessment (measurement). Describe comparability of assessment methods if there is more than one group | *3,4* | We used General Health Questionnaire to collect the basic information of all the participants.(…)We used X-ray, type-B ultrasonic to exclude possible organic lesions. Total serum cholesterol(TCHO), low density lipoprotein cholesterol (LDL-c), triglycerides(TG) and creatinine(CR) were detected by automatic biochemical analyzer(Hitachi, Japan). (…)Thebloodpressure was measured by physicians withastandardmercury sphygmomanometerwith a 14-cmcuff.(…)Lastly, we used reversed-phase HPLC fluorescence method(Shimadzu corporation, Kyoto, Japan) to determine the metabolites of MNTs in the urine of the participants.(…)The independent sample T-test or chi-square test was used to compare the above basic characteristics of two groups. |
| Bias | 9 | Describe any efforts to address potential sources of bias | 3,4 | Both groups ruled out these patients who had hematological, cardiovascular, respiratory or infectious related diseases. (…)measured by specially trained interviewers. (…) under standard operating procedures.(…)to estimate the crudeeffect of the exposure to blood pressures.(…) with adjusting for potential covariates. |
| Study size | 10 | Explain how the study size was arrived at | 3 | We conducted this survey in low level isopropyl alcohol exposed workers in one of sub-cohorts from this project in June 2013. |

Continued on next page

| Quantitative variables | 11 | Explain how quantitative variables were handled in the analyses. If applicable, describe which groupings were chosen and why | 4 | T-test or chi-square test was used to compare the differences of basic characteristics between two groups. (…)an entered multiple linear regression models were used to test the association between isopropyl alcohol exposure and blood pressures and to analyze the interaction effects of isopropyl alcohol exposure with characteristics (…)The mediating effects of the metabolites on the association between isopropyl alcohol exposure and blood pressures were determined with a series of linear regression models with adjusting for potential covariates. |
| --- | --- | --- | --- | --- |
| Statistical methods | 12 | (*a*) Describe all statistical methods, including those used to control for confounding | 4 | The independent sample T-test or chi-square test was used to compare the differences of basic characteristics between two groups. (…)multiple linear regression analysis to obtain the signiﬁcant covariates and to estimate the crude effect of the exposure to blood pressures. |
|  |  | (*b*) Describe any methods used to examine subgroups and interactions | 4 | an entered multiple linear regression models were used to test the association between isopropyl alcohol exposure and blood pressures and to analyze the interaction effects of isopropyl alcohol exposure with characteristics |
|  |  | (*c*) Explain how missing data were addressed | 5 | Value assignments and missing data in participants were shown inS1Table. |
|  |  | (*d*) *Cohort study*—If applicable, explain how loss to follow-up was addressed  *Case-control study*—If applicable, explain how matching of cases and controls was addressed  *Cross-sectional study*—If applicable, describe analytical methods taking account of sampling strategy | 4 | The values were expressed in the form of mean±SD(standard deviation) for the continuous indexes or the percent with number of cases for the categorical indexes. |
|  |  | (*e*) Describe any sensitivity analyses | 4 | We conducted a step by step multiple linear regression analysis to (…)and to estimate the crude effect of the exposure to blood pressures. (…) with adjusting for potential covariates. |
| Results | | | | |
| Participants | 13* | (a) Report numbers of individuals at each stage of study—eg numbers potentially eligible, examined for eligibility, confirmed eligible, included in the study, completing follow-up, and analysed | 5 | The study eventually collected 283 qualified samples (exposed200,controls83). |
|  |  | (b) Give reasons for non-participation at each stage |  |  |
|  |  | (c) Consider use of a flow diagram |  |  |
| Descriptive data | 14* | (a) Give characteristics of study participants (eg demographic, clinical, social) and information on exposures and potential confounders | 5 | The majority of all participants were young with the age range from 18 to 42 years old. The mean age of exposed group was (24.5±3.6) years and the control was (22.5±5.0) years. The isopropyl alcohol exposure years were (2.5±2.2). As expected, variables such as gender, nationality, birth place, marital status, income, smoking history, family history of CVDs in exposed group were comparable with controls. |
|  |  | (b) Indicate number of participants with missing data for each variable of interest | 5 | Value assignments and missing data in participants were shown inS1Table. |
|  |  | (c) *Cohort study*—Summarise follow-up time (eg, average and total amount) |  |  |
| Outcome data | 15* | *Cohort study*—Report numbers of outcome events or summary measures over time |  |  |
|  |  | *Case-control study—*Report numbers in each exposure category, or summary measures of exposure |  |  |
|  |  | *Cross-sectional study—*Report numbers of outcome events or summary measures | 5-8 | The concentrations of air isopropyl alcohol in all monitored workshops and time points were less than 100mg/m3 (see S2 Table).(…)Urinary acetone in exposed group was (3.0±5.3)mg/L, higher than that of control group(1.4±0.8) mg/L, the difference was significant (*p*< 0.05)(table1).(…)SBP, DBP and MBP were obviously associated with isopropyl alcohol exposure, the associations remained significant after adjusting for potential covariates (*p*<0.05). However, there was no difference when it came to PBP (*p*>0.05) (Table2). As shown in Fig.1, *(…)*significant interactions were observed between isopropyl alcohol exposure and smoking on DBP and MBP after adjusting for covariates (*p*_interaction_<0.05). (…) Fig.2 illustrates the mediation effect and shows that isopropyl alcohol exposure is significantly associated with DBP(c=0.171, *p*<0.05) and MBP(c=0.163, *p*<0.05) after adjusting for the potential covariates. |
| Main results | 16 | (*a*) Give unadjusted estimates and, if applicable, confounder-adjusted estimates and their precision (eg, 95% confidence interval). Make clear which confounders were adjusted for and why they were included | 6-7 | **Table 2Blood pressures (mmHg) in isopropyl alcohol exposed workers and controls**   | **Blood pressure** | **Exposed** | **Controls** | **D-value** | | ***P*_crude_** | ***P*_ad._** | | --- | --- | --- | --- | --- | --- | --- | | **SBP, mean±SD** | 123±10 | 118±11 | 5 | 0.003 | | 0.012 | | **DBP, mean±SD** | 79±7 | 74±7 | 5 | 0.001 | | 0.005 | | **MBP, mean±SD** | 93±8 | 89±9 | 4 | 0.001 | | 0.008 | | **PBP, mean±SD** | 44±7 | 44±8 | 0 | 0.725 | | 0.962 |   SD: standard deviation.SBP: Systolic Blood Pressure. DBP: diastolic blood pressure. MBP: mean blood pressure. PBP: pulse blood pressure.D-value: the mean difference between exposed and controls.*p*_crude_: unadjusted for covariates. *p*_ad_: adjusted for potential covariates. |
|  |  | (*b*) Report category boundaries when continuous variables were categorized |  |  |
|  |  | (*c*) If relevant, consider translating estimates of relative risk into absolute risk for a meaningful time period |  |  |

Continued on next page

| Other analyses | 17 | Report other analyses done—eg analyses of subgroups and interactions, and sensitivity analyses | 7-8 | Significant interactions were observed between isopropyl alcohol exposure and smoking on DBP and MBP after adjusting for covariates (*p*_interaction_<0.05).(…)5-HIAA mediated the association between isopropyl alcohol exposure and DBP, MBP. The meditative effects on DBP and MBP can be expressed as(-0.338 × -0.120)/[(-0.338 × -0.120) + 0.134] = 23.1% and (-0.338 × -0.124)/[( -0.338 × -0.124) + 0.124] = 25.3%, respectively. |
| --- | --- | --- | --- | --- |
| Discussion | | | | |
| Key results | 18 | Summarise key results with reference to study objectives | 9 | To our knowledge, this study provided first evidence that low level isopropyl alcohol exposure was associated with increased blood pressure. Previous studies found that high concentrations of isopropyl alcohol can reduce blood pressure, leading to hypotension in isopropyl alcohol poisoning population. Here we found that low level isopropyl alcohol could increase arterial blood pressures. This phenomenon may share a special effect called “hormesis” which can be observed in many chemicals at low level exposure.In addition, we observed an interaction between isopropyl alcohol exposure and smoking and discovered a mediating role of 5-HIAA on low level isopropyl alcohol exposure induced increase of blood pressures, which might shed light for further mechanism studies. |
| Limitations | 19 | Discuss limitations of the study, taking into account sources of potential bias or imprecision. Discuss both direction and magnitude of any potential bias | 9,10 | We analyzed the relationship between the different exposure levels and blood pressure according to the concentration of acetone in urine. Although it suggested an increased tendency, we have not provided formal proof yet that there is a dose - response relationship between them. This may be related to the narrow range of exposure level itself. Another possible reason is that other individual difference such as genetic factors, behavior and life style interact with low level isopropyl alcohol exposure, leading to the modified results. |
| Interpretation | 20 | Give a cautious overall interpretation of results considering objectives, limitations, multiplicity of analyses, results from similar studies, and other relevant evidence | 11 | One may conclude that low level isopropyl alcohol exposure was associated with increased blood pressure. Besides, our work supported the view of chemical hormesis and tested our hypothesis of its effect on blood pressures. We demonstrated that the interaction effect of isopropyl alcohol and smoking on blood pressure and found the mediation effect of 5-HIAA on the association between isopropyl alcohol exposure and blood pressures based on an occupational population study. More studies are needed to fully study the cardiovascular toxicity and mechanism of isopropyl alcohol and its interaction with other risk factors. |
| Generalisability | 21 | Discuss the generalisability (external validity) of the study results | 9 | Our exposure assessment showed that the exposed subjects had a low level of isopropyl alcohol exposure. On the one hand, we evaluated isopropyl alcohol concentration in the air of working environment by reviewing the monitored data in last 4 years. On the other hand, we detected the individual internal exposure level through urine. Both of the results were far less than the exposure limit. But they are greater than that in controls. Other organic components in the workplace, including benzene, acetone, xylene, toluene and methanol were not detectable (not mentioned), which suggesting that isopropyl alcohol was the main occupational risk factor. |
| Other information | |  | | |
| Funding | 22 | Give the source of funding and the role of the funders for the present study and, if applicable, for the original study on which the present article is based | 11 | This research was supported by the Natural Science Foundation of China (No.81273097，81472998) and by the One Hundred Talents Program of Sun Yat-senUniversity (No. 18801031). |

*Give information separately for cases and controls in case-control studies and, if applicable, for exposed and unexposed groups in cohort and cross-sectional studies.

**Note:** An Explanation and Elaboration article discusses each checklist item and gives methodological background and published examples of transparent reporting. The STROBE checklist is best used in conjunction with this article (freely available on the Web sites of PLoS Medicine at http://www.plosmedicine.org/, Annals of Internal Medicine at http://www.annals.org/, and Epidemiology at http://www.epidem.com/). Information on the STROBE Initiative is available at [www.strobe-statement.org](http://www.strobe-statement.org/).
