# Supplementary material for: The Activation Effects of Low Level Isopropyl Alcohol Exposure on Arterial Blood Pressures Are Associated with Decreased 5-Hydroxyindole Acetic Acid in Urine
Source: PLoS One. 2016 Sep 13;11(9):e0162762. doi: 10.1371/journal.pone.0162762 (PMC5021351; doi:10.1371/journal.pone.0162762)
Supplement: S1 Table — (DOC) [file pone.0162762.s003.doc]

**S1 table** Value assignments and missing data in this study

| **Characteristic** | **assignments value** | **Exposed group %(N)** | **Controls % (N)** |
| --- | --- | --- | --- |
| **Sex** |  | 0.0(0) | 0.0(0) |
| **male** | 1 |  |  |
| **female** | 2 |  |  |
| **Age(years)** |  | 0.0(0) | 0.0(0) |
| **<20** | 1 |  |  |
| **20-30** | 2 |  |  |
| **≥30** | 3 |  |  |
| **BMI(kg/m2)** |  | 4.0(8) | 0.0(0) |
| **<24** | 1 |  |  |
| **24-28** | 2 |  |  |
| **≥28** | 3 |  |  |
| **Waist circumstance(cm)** |  | 2.5(5) | 1.2(1) |
| **<85(M),<80(F)** | 1 |  |  |
| **≥85(M),≥80(F)** | 2 |  |  |
| **Race** |  | 2.5(5) | 0.0(0) |
| **han** | 1 |  |  |
| **others** | 2 |  |  |
| **Birth place** |  | 2.5(5) | 0.0(0) |
| **Town** | 1 |  |  |
| **Village** | 2 |  |  |
| **Highest school level** |  | 1.0(2) | 0.0(0) |
| **Primary** | 1 |  |  |
| **Technical** | 2 |  |  |
| **College or above** | 3 |  |  |
| **Marital status** |  | 1.5(3) | 2.4(2) |
| **Married** | 1 |  |  |
| **Unmarried** | 2 |  |  |
| **Others** | 3 |  |  |
| **Income(yuan)** |  | 1.5(3) | 4.8(4) |
| **≤2000** | 1 |  |  |
| **≤4000** | 2 |  |  |
| **≤6000** | 3 |  |  |
| **＞6000** | 4 |  |  |
| **Smoking history** |  | 8.5(17) | 0.0(0) |
| **Yes** | 1 |  |  |
| **No** | 0 |  |  |
| **Drinking history** |  | 5.5(11) | 1.2(1) |
| **Yes** | 1 |  |  |
| **No** | 0 |  |  |
| **Family history of CVDs** |  | 7.0(14) | 1.2(1) |
| **Yes** | 1 |  |  |
| **No** | 0 |  |  |
| **isopropyl alcohol contact(years)** | - | 0.0(0) | - |
| **Working time(hours)** | - | 0.0(0) | 0.0(0) |
| **LDL-C(ug/g CR)** | - | 0.0(0) | 0.0(0) |
| **TG(ug/g CR)** | - | 0.0(0) | 0.0(0) |
| **TCHO(ug/g CR)** | - | 0.0(0) | 0.0(0) |
| **Urinary acetone(mg/L)** | - | 0.0(0) | 0.0(0) |
| **Urinary creatinine(mg/L)** | - | 0.0(0) | 0.0(0) |
| **SBP(mmHg)** | - | 0.5(1) | 3.6(3) |
| **DBP(mmHg)** | - | 0.5(1) | 3.6(3) |
| **VMA(mg/g CR)** | - | 1.0(2) | 0.0(0) |
| **DOPAC(mg/g CR)** | - | 1.0(2) | 0.0(0) |
| **5-HIAA(mg/g CR)** | - | 1.0(2) | 0.0(0) |
| **HVA(mg/g CR)** | - | 1.0(2) | 0.0(0) |
