# Supplementary material for: The Activation Effects of Low Level Isopropyl Alcohol Exposure on Arterial Blood Pressures Are Associated with Decreased 5-Hydroxyindole Acetic Acid in Urine
Source: PLoS One. 2016 Sep 13;11(9):e0162762. doi: 10.1371/journal.pone.0162762 (PMC5021351; doi:10.1371/journal.pone.0162762)
Supplement: S2 Table — (DOC) [file pone.0162762.s004.doc]

**S2 Table** Isopropyl alcohol concentration (mg/m3) in the air of workshop

| **Workplace** | | | | |
| --- | --- | --- | --- | --- |
| **Category** | **Check point 1** | **Check point 2** | **Check point 3** | **Check point 4** |
| **Year 1** | 4.8 | 1.7 | 2.3 | 1.8 |
| **Year 2** | 83.6 | 10 | 24.4 | - |
| **Year 3** | 154.1 | 26.7 | 53 | 28.8 |
| **Year 4** | 50.7 | 35.5 | 36.1 | 25.3 |
| **Mean±SD** | 73.3±62.8 | 18.5±15.4 | 29.0±21.3 | 18.6±14.7 |
