# Supplementary material for: The Activation Effects of Low Level Isopropyl Alcohol Exposure on Arterial Blood Pressures Are Associated with Decreased 5-Hydroxyindole Acetic Acid in Urine
Source: PLoS One. 2016 Sep 13;11(9):e0162762. doi: 10.1371/journal.pone.0162762 (PMC5021351; doi:10.1371/journal.pone.0162762)
Supplement: S3 Table — (DOC) [file pone.0162762.s005.doc]

**S3 table** Potential covariates of arterial blood pressures among basic characteristics

| coefficient ^a^ | | | | | | | | | | | | | | | | |
| --- | --- | --- | --- | --- | --- | --- | --- | --- | --- | --- | --- | --- | --- | --- | --- | --- |
| **Models** | | | | **Unstandardized Coefficients** | | | | **standardized Coefficients** | | | | | ***t*** | | ***p*** | |
|  |  |  |  | **B** | | **Standard error** | |  | | | | |  |  |  |  |
| 1 | | Constant | | 93.472 | | 5.540 | | | |  | 16.872 | | | | .000 | |
|  |  | BMI | | 1.339 | | .264 | | | | .352 | 5.066 | | | | .000 | |
| 2 | | constant | | 102.556 | | 6.443 | | | |  | 15.916 | | | | .000 | |
|  |  | BMI | | 1.242 | | .263 | | | | .326 | 4.727 | | | | .000 | |
|  |  | Birth place | | -4.097 | | 1.550 | | | | -.182 | -2.644 | | | | .009 | |
| a. dependent variable: SBP. BMI: body mass index. | | | | | | | | | | | | | | | | |
| coefficient ^b^ | | | | | | | | | | | | | | | | |
| **Models** | | | | | **Unstandardized Coefficients** | | | | **standardized Coefficients** | | | | ***t*** | | | ***p.*** |
|  |  |  |  |  | **B** | | **Standard error** | |  | | | |  |  |  |  |
| 1 | Constant | | | | 63.963 | | 3.145 | |  | | | | 20.338 | | | .000 |
|  | Age | | | | .570 | | .132 | | .304 | | | | 4.305 | | | .000 |
| 2 | Constant | | | | 52.665 | | 4.846 | |  | | | | 10.868 | | | .000 |
|  | Age | | | | .438 | | .137 | | .234 | | | | 3.206 | | | .002 |
|  | Waist circumstance | | | | .191 | | .063 | | .220 | | | | 3.018 | | | .003 |
| 3 | Constant | | | | 46.084 | | 5.423 | |  | | | | 8.498 | | | .000 |
|  | Age | | | | .446 | | .135 | | .238 | | | | 3.310 | | | .001 |
|  | Waist circumstance | | | | .209 | | .063 | | .241 | | | | 3.337 | | | .001 |
|  | Race | | | | 4.553 | | 1.780 | | .176 | | | | 2.558 | | | .011 |
| 4 | Constant | | | | 47.096 | | 5.397 | |  | | | | 8.726 | | | .000 |
|  | Age | | | | .459 | | .134 | | .245 | | | | 3.434 | | | .001 |
|  | Waist circumstance | | | | .191 | | .063 | | .220 | | | | 3.037 | | | .003 |
|  | Race | | | | 4.224 | | 1.772 | | .163 | | | | 2.384 | | | .018 |
|  | Drinking | | | | 3.241 | | 1.574 | | .141 | | | 2.059 | | | | .041 |
| b. dependent variable: DBP. | | | | | | | | | | | | | | | | |
| coefficient c | | | | | | | | | | | | | | | | |
| **Models** | | | **Unstandardized Coefficients** | | | | | **standardized Coefficients** | | | | | | *t* | | *p* |
|  |  |  | B | | | **Standard error** | |  | | | | | |  |  |  |
| 1 | Constant | | 70.031 | | | 4.632 | |  | | | | | | 15.119 | | .000 |
|  | Waist circumstance | | .291 | | | .061 | | .333 | | | | | | 4.769 | | .000 |
| 2 | Constant | | 77.262 | | | 5.317 | |  | | | | | | 14.531 | | .000 |
|  | Waist circumstance | | .270 | | | .061 | | .309 | | | | | | 4.447 | | .000 |
|  | Birth place | | -3.254 | | | 1.232 | | -.183 | | | | | | -2.641 | | .009 |
| 3 | Constant | | 72.891 | | | 5.592 | |  | | | | | | 13.035 | | .000 |
|  | Waist circumstance | | .225 | | | .063 | | .258 | | | | | | 3.577 | | .000 |
|  | Birth place | | -3.004 | | | 1.223 | | -.169 | | | | | | -2.456 | | .015 |
|  | Age | | .311 | | | .136 | | .165 | | | | | | 2.290 | | .023 |
| c. dependent variable: MBP | | | | | | | | | | | | | | | | |

| coefficient ^d^ | | | | | | |
| --- | --- | --- | --- | --- | --- | --- |
| **models** | | **Unstandardized Coefficients** | | **standardized Coefficients** | *t* | *p* |
|  |  | B | **Standard error** |  |  |  |
| 1 | constant | 31.811 | 4.240 |  | 7.502 | .000 |
|  | BMI | .588 | .202 | .210 | 2.904 | .004 |
| 2 | constant | 36.309 | 4.613 |  | 7.871 | .000 |
|  | BMI | .711 | .207 | .255 | 3.439 | .001 |
|  | age | -.302 | .130 | -.173 | -2.330 | .021 |
| d. dependent variable: PBP | | | | | | |
