# Supplementary material for: The Activation Effects of Low Level Isopropyl Alcohol Exposure on Arterial Blood Pressures Are Associated with Decreased 5-Hydroxyindole Acetic Acid in Urine
Source: PLoS One. 2016 Sep 13;11(9):e0162762. doi: 10.1371/journal.pone.0162762 (PMC5021351; doi:10.1371/journal.pone.0162762)
Supplement: S4 Table — (DOC) [file pone.0162762.s006.doc]

**S4 table** Exposure-response relationship between urinary acetone concentrations among workers exposed to low level isopropyl alcohol and controls, and arterial blood pressures(mmHg)^*^

| **Category** | **exposed** | | | | **controls** | | ^＃^***P*_trend_(all)** | | ^§^***P*_trend_(exposed.)** | |
| --- | --- | --- | --- | --- | --- | --- | --- | --- | --- | --- |
|  | **Q1** | **Q2** | **Q3** | **Q4** |  |  |  |  |  |  |
| SBP | 123±10 | 124±10 | 122±10 | 122±12 |  | 118±11 | | 0.831 | | 0.707 |
| DBP | 80±7 | 79±6 | 78±7 | 78±9 |  | 74±7 | | 0.384 | | 0.817 |
| MBP | 94±8 | 94±7 | 93±8 | 93±9 |  | 89±9 | | 0.813 | | 0.974 |
| PBP | 43±6 | 46±7 | 44±6 | 44±8 |  | 44±8 | | 0.487 | | 0.435 |

*Workers were classified into four groups according to the quartiles of urinary acetone concentrations (ug/ml)(Q1: <1.135, Q2: 1.135-1.470, Q3: 1.470-2.675, Q4: ≥2.675).

^#^*P*: category of low level isopropyl alcohol exposure (urinary acetone concentrations) as an ordinal variable taking on values of 0,1, 2, 3 and 4(controls,Q1, Q2,Q3 and Q4), adjusted for potential covariates.

^§^*P*: category of low level isopropyl alcohol exposure (urinary acetone concentrations) as an ordinal variable taking on values of 1, 2, 3 and 4(Q1, Q2, Q3 and Q4), adjusted for potential covariates..
