# Supplementary material for: The Activation Effects of Low Level Isopropyl Alcohol Exposure on Arterial Blood Pressures Are Associated with Decreased 5-Hydroxyindole Acetic Acid in Urine
Source: PLoS One. 2016 Sep 13;11(9):e0162762. doi: 10.1371/journal.pone.0162762 (PMC5021351; doi:10.1371/journal.pone.0162762)
Supplement: S5 Table — (DOC) [file pone.0162762.s007.doc]

**S5 table** The metabolites of monoamine neural transmitters in isopropyl alcohol exposed workers and controls

|  | **Exposed** | **Controls** | **p_crude._** | **p_ad._** |
| --- | --- | --- | --- | --- |
| VMA(mg/g CR), mean ±SD | 5.1±2.1 | 8.6±12.6 | <0.001 | <0.001 |
| DOPAC(mg/gCR), mean ±SD | 14.6±9.8 | 25.7±33.9 | 0.004 | <0.001 |
| 5-HIAA(mg/gCR), mean ±SD | 2.8±1.9 | 5.6±7.6 | <0.001 | <0.001 |
| HVA(mg/g CR), mean ±SD | 4.2±3.8 | 4.3±5.9 | 0.835 | 0.908 |

SD: standard deviation. VMA: vanillylmandelic acid. DOPAC: dihydroxyphenylacetic acid. 5-HIAA: 5-hydroxy indole acetic acid. HVA: homovanillic acid. CR: creatinine. *p_crude_*: unadjusted for covariates.  *p_ad_.*: adjusted for potential covariates.
